# Wandering Carotid Artery in Carotid Artery Stenting

**DOI:** 10.7759/cureus.78788

**Published:** 2025-02-09

**Authors:** Atsushi Ogata, Takashi Furukawa, Jun Masuoka, Tatsuya Abe

**Affiliations:** 1 Neurosurgery, Saga University, Saga, JPN; 2 Neurosurgery, Faculty of Medicine, Saga University, Saga, JPN

**Keywords:** carotid artery, magnetic resonace imaging, stenosis, stenting, wandering

## Abstract

A wandering carotid artery is the rare phenomenon of the repeated migration of the carotid artery into the lateral and retropharyngeal spaces. We report a case of wandering carotid artery before and after carotid artery stenting (CAS). The patient was an 82-year-old female with symptomatic right internal carotid artery (ICA) stenosis. A diagnostic digital subtraction angiography (DSA) showed that while the positional relationship between the internal and external carotid arteries was typical, the right ICA had moved medially into retropharyngeal space during CAS. Magnetic resonance angiography a day after CAS showed that the ICA had returned to its original position.

## Introduction

A retropharyngeal carotid artery (RCA) is an anatomical variation where the carotid artery resides in the retropharyngeal space. It is considered relatively rare, with an estimated incidence of 2.6% in the general population [1]. The carotid artery may move into the retropharyngeal space in a relatively short period [2,3]. A wandering carotid artery is the rare phenomenon of the repeated migration of the carotid artery into the lateral and retropharyngeal spaces [4,5]. We report a case of wandering carotid artery before and after carotid artery stenting (CAS).

## Case presentation

**Figure 1:**
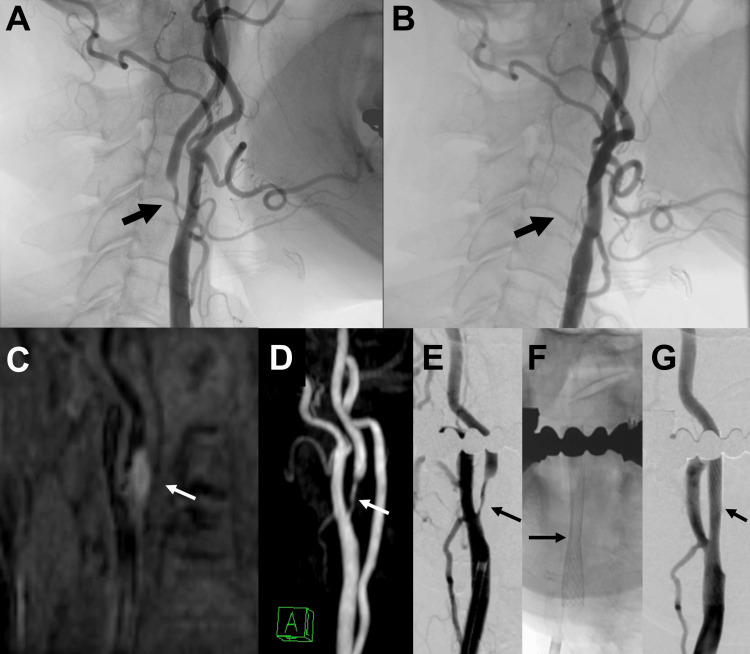
Angiographic images demonstrating carotid artery position changes (A) Lateral right carotid angiography 2 months before carotid artery stenting (CAS) shows severe stenosis of the right internal carotid artery (ICA). (B) Lateral right carotid angiography of CAS shows that the external and internal carotid arteries overlap, and the carotid bifurcation cannot be seen. (C) Coronal magnetization-prepared rapid acquisition gradient-echo (MPRAGE) imaging shows high signal intensity plaque along the medial origin of the right ICA. (D) Time-of-flight magnetic resonance angiography (TOF-MRA) on the day before CAS clearly shows carotid bifurcation in the left anterior oblique (LAO) 15° projection. (E, F, G) LAO 15° was selected as a working projection during CAS. A stent (Carotid Wallstent 10/24) was deployed after predilation under filter protection.

**Figure 2:**
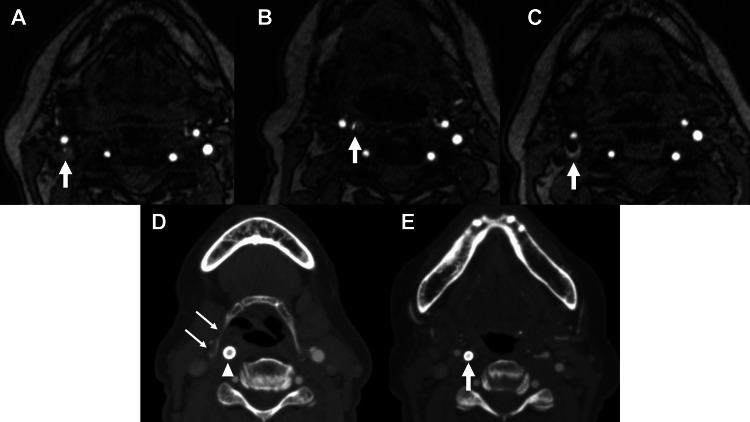
Serial imaging studies showing dynamic position changes of the internal carotid artery before and after stenting Time-of-flight magnetic resonance angiography (TOF-MRA) 2 months before (A), a day before (B), and a day after (C) carotid artery stenting (CAS). The internal carotid artery (ICA) moved into the retropharyngeal space in the MRA the day before CAS and returned to its original position the day after CAS. The arrow indicates the ICA. (D, E) Computed tomography angiography (CTA) 5 days after CAS. The common carotid artery (arrowhead) is medial to the hyoid bone (double arrows), and the ICA (arrow) has also moved medially again.

The patient is an 82-year-old woman with hypertension and dyslipidemia, and no history of neck trauma, surgery, or radiation. She had a transient ischemic attack and was diagnosed with symptomatic right internal carotid artery (ICA) stenosis as a result of examinations, including diagnostic digital subtraction angiography (DSA). In the diagnostic DSA, the positional relationship between the internal and external carotid arteries was typical, and the carotid bifurcation could be clearly observed in the lateral view (Figure 1A). In the anteroposterior view, the internal and external carotid arteries overlapped. Two months after the examination, the patient underwent CAS. However, during CAS, the external and internal carotid arteries overlapped in the lateral view, and the carotid bifurcation could not be clearly observed (Figure 1B). Coronal magnetization-prepared rapid acquisition gradient-echo (MP-RAGE) (Figure 1C) and time-of-flight magnetic resonance angiography (TOF-MRA) imaging (Figure 2A, 2B) on the day before CAS showed that the right ICA had moved medially, resulting in an RCA. On MP-RAGE, high signal intensity plaque was observed along the medial origin of ICA, suggesting plaque instability (Figure 1C). Based on TOF-MRA, a left anterior oblique angle of 15° was selected as a working projection during CAS (Figure 1D, 1E). The Carotid Wallstent 10/24 (Boston Scientific, Natick, Massachusetts, USA) was deployed after predilation under filter protection (Figure 1F). The position of the ICA did not change on angiography immediately after CAS (Figure 1G). MRA a day after CAS showed that the ICA had returned to its original position (Figure 2C). Five days after CAS, computed tomography angiography (CTA) showed that the ICA had again moved into the retropharyngeal space, and the common carotid artery (CCA) was located medial to the hyoid bone (Figure 2D, 2E). The patient did not experience any complications. No restenosis or stroke events were observed during the 42-week follow-up period after CAS.

## Discussion

It is not uncommon for the carotid artery to migrate medially over a short period, resulting in RCA. In a study using contrast-enhanced computed tomography of patients without cervical involvement, a moving carotid artery was found in 4 of 63 patients (6.3%) [2]. All patients presented with RCA at the level of the hyoid bone [2]. In our case, the carotid artery was also located medial to the hyoid bone when the carotid artery became RCA.

Dynamic magnetic resonance imaging studies have shown that the carotid artery can move anteromedially with a swallowing motion [6]. In our case, it is assumed that the CCA was moved anteromedially by a swallowing motion and then fixed to the hyoid bone, resulting in an RCA. It is possible that the positional relationship between the internal and external carotid arteries changed accordingly. An elongated hyoid bone can cause mechanical compression of ICA, leading to dissection and atherosclerotic stenosis [7,8]. Therefore, the longer hyoid bone may be more prone to a wandering carotid artery. RCA can be classified into two types: dynamic wandering carotid artery as in our case, and static RCA resulting from developmental anomalies or arterial tortuosity. While RCA is often asymptomatic, some patients may experience dysphagia, foreign body sensation, or rarely, pulsatile mass sensation in the pharynx. In our case, the patient was asymptomatic regarding the RCA position. The exact pathophysiological mechanisms require further investigation through additional case studies.

The optimal treatment strategy for managing a stenotic wandering carotid artery, namely carotid endarterectomy (CEA) versus CAS, remains undetermined due to limited reported cases [2,3]. In CEA, surgical exploration may be technically challenging with retropharyngeal carotid artery positioning. For CAS, vessel mobility could theoretically lead to complications such as stent migration or incomplete wall apposition when using self-expandable stents. However, our case demonstrated successful CAS implementation and no stent-related complications during the 42-week follow-up period. Further research is needed to evaluate the effects of continued vessel movement on stent endothelialization and long-term outcomes.

No case of CAS with moving or wandering carotid artery has been previously reported. However, the possibility of encountering a moving or wandering carotid artery should also be considered before and after CAS.

## Conclusions

A wandering carotid artery can coexist with carotid artery stenosis. Our case demonstrated that CAS can be successfully performed in a wandering carotid artery with good mid-term outcomes, though the long-term effects of a wandering carotid artery require further investigation.
